# Factors influencing choice of site for rural clinical placements by final year medical students in a South African university

**DOI:** 10.4102/phcfm.v9i1.1226

**Published:** 2017-04-28

**Authors:** Nontsikelelo O. Mapukata, Rainy Dube, Ian Couper, Motlatso G. Mlambo

**Affiliations:** 1 Centre for Rural Health, Faculty of Health Sciences, University of the Witwatersrand, South Africa; 2 Ukwanda Centre for Rural Health, Faculty of Health Sciences, Stellenbosch University, South Africa

## Abstract

**Background:**

Most of South Africa’s citizens who live in rural or underserved communities rely on the public health care sector to access quality health care. The value of rural exposure through clinical placements is well documented. Medical schools in South Africa have a responsibility to provide solutions that address the prevailing human resources challenges. Despite this commitment, medical students do not necessarily appreciate their role in resolving South Africa’s human resources challenges. This study aimed to assess the factors that influenced the choice of clinical learning sites in a self-selection process undertaken by Wits final year medical students for the compulsory 6-week integrated primary care block rotation.

**Methods:**

Qualitative data related to reasons for choice of service learning site were gathered from 524 pre-placement questionnaires completed by final year medical students entering the rotation over a 3-year period (2012–2014). Thematic analysis was performed using the MAXQDA software.

**Results:**

Eight themes emerged from the study indicating that the majority of participants were in favour of local urban underserved placement. Contextual factors, such as work commitments or family responsibilities, being compromised socially and losing academic standing were the main reasons for seeking urban placement. Good supervision, opportunistic learning, skills development and moral support were reasons for seeking rural placements. Previous voluntary exposure to rural practice or being of rural origin was a strong indicator for uptake of rural placement.

**Conclusion:**

This study has demonstrated the challenges faced by coordinators in balancing personal and institutional needs with country needs and the contextual factors that must be considered when implementing medical education programmes that respond to social challenges.

## Introduction

South Africa is a developing country with many of its citizens residing in rural or underserved communities dependent on the public health care sector to access quality health care. Universities across the country are tasked with the responsibility to provide solutions for prevailing challenges such as the shortage of health care workers, especially in the rural areas of South Africa.^1,2^ In the first 20 years of post-apartheid, South African universities had an opportunity to select a diverse group of health sciences students and adopt curricula that focused on reforms to benefit communities.^3,4^ Reflecting on some of these initiatives, Lehmann et al. refer to a full spectrum of reforms that brought changes and innovations in many of the medical schools.^5^ Although these initiatives varied from institution to institution, from student selection as an entry point right up to faculty development to incorporate a diverse body of staff members, the ultimate goal has always been to produce a graduate who will respond to the needs of South Africa. It is for these reasons that rural exposure through clinical placements has gained prominence amongst policy makers, faculty members and clinical educators based on perceived benefits in familiarising students with the needs of rural communities and an opportunity to address the cumulative shortage of health care workers.^6,7,8^ The question remains, however, whether these initiatives are having the impact that is desired, and whether medical students are changing in their attitudes to the perceived needs.

Research has shown that being of rural origin is positively associated with having intentions to work in a rural community.^6,7,9^ There is a body of work suggesting that rural clinical placements for medical students could create interest in working in a rural setting.^4,6,10^ A positive relationship between such placements and later work in rural areas has also been found.^6,11,12^ Amongst the many reforms adopted by the Faculty of Health Sciences at the University of the Witwatersrand (Wits) was the launch of the Graduate Entry Medical Programme (GEMP) in 2003 as a 4-year training programme that complemented the existing traditional approach to medical training, with both streams being combined in the clinical years. Within that, the integrated primary care (IPC) block, which is a compulsory 6-week placement in a range of primary health care settings, was launched in 2006 as one of the initiatives that would strengthen the university’s and students’ commitment to rural and underserved communities.^8^ Through the IPC block, district facilities in underserved communities of the Gauteng, North West and Mpumalanga provinces provide the context for final year medical students to achieve some of the core competencies of an integrated curriculum. This allows students to understand not only the disease profile but also the health care needs of that particular community.^8,12^ Students are able to select the sites at which they wish to undergo the IPC rotation in small groups of 3–4 within a range of rural, peri-urban and urban sites offered for each block, comprising 30 to 36 students. The reasons for their choices of site are unknown.

This study aimed to assess factors that influenced the choice of sites in the self-selection process undertaken by final year medical students at Wits for the compulsory 6-week IPC block in the period 2012–2014.

## Methodology

### Setting

In preparation for the IPC block placement, final year medical students are required to attend an hour’s briefing session on a Friday afternoon 4 weeks prior to the rotation. Students are briefed on the objectives of the block, site availability and the number of students that can be accommodated in each site. Following the briefing, 30–36 final year medical students have an opportunity to choose where they wish to be based from any of the 7–9 sites available to host students per rotation. Students have to negotiate their choice with their peers, taking into account issues such as students who have special needs, such as personal or family circumstances or having to work to pay tuition fees. Academic coordinators are required to support special case requests from the Office of Student Support, such as a student who requires remediation or is ill.

As part of the IPC block orientation on the first day of each rotation, students complete a pre-placement questionnaire that evaluates their expectations of the block.

### Sample and data collection

A total of 524 final year students completed the pre-placement questionnaire consisting of open- and closed-ended questions over a 3-year period from 2012 to 2014. This study focused only on the qualitative part of the questionnaire, specifically student responses to an open-ended question, ‘What factors influenced the choice of site you will be going to?’

### Data analysis

Thematic content analysis was performed using the MAXQDA software (Version 11). This provided a detailed account of participants’ responses. An inductive analysis approach was used to generate codes and identify themes. The initial analysis was conducted by M.G.M. and was finalised by N.O.M. with consensus from the co-authors. Informed consent was obtained from all students who participated. The data were anonymised and confidentiality was maintained throughout the research process.

## Results

The majority of participants were in favour of urban underserved local placements in Gauteng province. In terms of the factors influencing choice of site, eight themes emerged from the study. These are reported below under the two subheadings as factors that led to interest in either rural or urban placements. The researchers excluded 19 students’ responses in the final analysis as their allocation to rural sites was for space reasons and not in keeping with their stated preference for urban sites. Their comments did not fit either of the main themes as they focused on the fact that they did not get their choice.

### Demographics

Female students constituted 63% (325) of the total population. The large majority of the students (477; 91%) reported themselves to be South African citizens. At the time of completing the questionnaire, 309 (59%) students were aged between 20 and 24 years. Students were admitted mostly as undergraduates, at MBBCh 1 level (299; 64%) with most of them indicating no prior work experience (250; 53%) (see Table 1). Only 21% of the class reported themselves to be of rural origin.

**TABLE 1:** Participants’ demographic characteristics.

Variable	*N*	%
Gender		
Male	190	37.0
Female	325	63.0
Age (years)		
Below 20	4	0.8
20–24	309	59.0
25–29	186	35.5
30 and above	25	5.0
Population		
African	209	40.4
White	184	35.6
Coloured	27	5.2
Indian	81	16.0
Other	16	3.1
South African citizen		
Yes	477	91.0
No	47	9.0
Level of admission to Wits		
Entry to MBBCH1	299	64.0
Entry to GEMP1 [MBBCH III]	165	35.6
Number of years at Wits University		
1–4	46	9.7
5–6	247	52.0
7–8	142	30.0
More than 8	40	8.4
Work experience (year)		
No work experience	250	53.0
<1	95	20.0
1–4	99	21.0
5–10	25	5.0
More than 10	1	0.2

## Factors leading to interest in rural placements

The three themes that were mentioned by students who prioritised rural placement, were academic learning, interest in rural practice and rural environment.

### Academic learning in a rural facility

Amongst the reasons that influenced the students to choose a rural site were good supervision and also the ability to complete the requirements of the block.

‘I went to the place [*site*] briefly last year in GEMP 3 and saw a place that would teach me a lot in primary health care.’ (Participant 205, rotation 7, 2013)

‘Rural experience. Medicine outside of academic circuit.’ (Participant 103, rotation 3, 2013)

‘I want to be in rural area with smaller health care facilities. I think working where there is slightly fewer senior doctors provide an environment where students are ”forced” to learn and implement.’ (Participant 201, rotation 7, 2013)

Students also cited professional development as having an important influence.

‘The range of clinical scenarios I am likely to encounter. Working in a non-academic hospital to learn and work with colleagues in an expected way (i.e. maintaining punctuality, confidentiality) etc. and respect for people I will be working with.’ (Participant 68, rotation 3, 2013)

This development occurs because rural facilities provide an opportunity for students to assume responsibility for their patients and engage in teamwork.

‘The rural experience. The responsibility as a first site doctor. To better relationship with the group I work with.’ (Participant 90, rotation 4, 2013)

‘Wanting to work in a rural facility where staff is limited so hopefully I can be of value.’ (Participant 43, rotation 2, 2014)

‘Throwing myself in the deep end. Away from family and having to rely on myself and other team members. I’m hoping I will build confidence in myself and my capabilities.’ (Participant 37, rotation 2, 2014)

Managing the undifferentiated patient, a key outcome of the rotation, was cited a number of times in relation to skills acquisition and the corresponding professional responsibility in relation to the context.

‘Working at the clinics, seeing undifferentiated patients it will be so exciting to see if I can truly make decisions about patient’s pathology and management.’ (Participant 95, rotation 3, 2013)

The role of the primary health care setting was also relevant, in relation to the set outcomes of the medical curriculum.

‘Previous groups that has been there and had good experiences. Wanted an experience outside JHB, to experience how things work outside Gauteng.’ (Participant 129, rotation 4, 2014)

‘I wanted to experience primary health care in a rural setting.’ (Participant 152, rotation 5, 2013)

‘Experiences and advice from previous students. Also choosing a rural setting for the IPC Block in important, i.e. so we can become aware of the diversity and social differences within SA.’ (Participant 74, rotation 2, 2012)

### Interest in rural practice

Previous exposure to the facility was a strong motivator for repeated experience, as well as a commitment to rural practice.

‘I was exposed to rural medicine during my GEMP 1[MBBCh III] elective, I loved it and I am hoping to have a similar experience.’ (Participant 23, rotation 1, 2014)

‘I wanted to spend my IPC rotation in a rural area and having spent time at Tintswalo hospital [*a rural Mpumalanga province*] – I wanted to go back.’ (Participant 92, rotation 3, 2012)

A few students considered the relevance of the experience, rural origin and the exposure to their future plans.

‘I am interested in rural health, also I heard from previous students that Lehurutshe [*a rural North West hospital*] is a good site to be at for IPC.’ (Participant 9, rotation 1, 2014)

‘I would like to go back to North West for internship as I grew up in the area, would like to be part of the North West Department of Health setting.’ (Participant 126, rotation 4, 2013)

Others also considered the added benefit to communicate with patients in their home language.

‘Language issues – I speak Setswana and would like to work with Setswana speaking people to improve my efficacy as we do most learning in English.’ (Participant 151, rotation 4, 2012)

‘Rural experience. Learning to deal with the language barrier, beautiful surroundings.’ (Participant 96, rotation 3, 2013)

A number of students cited that rural exposure provides an opportunity for clinical practice, managing problems independently and ability to integrate their learning from GEMP 1 (MBBCh III) to GEMP 4 (MBBCh VI) in relation to the requirements of the profession.

‘I am excited to see how well I have integrated my clinical knowledge. I want to manage patients from the beginning and follow them up. I enjoy primary healthcare.’ (Participant 31, rotation 1, 2013)

‘Starting to be a doctor! To learn as much of clinical practice as possible and to practice biopsychosocial approach … To learn management of basic problems and to work by myself for the 1st time … to integrate all the medical teaching I have received and gain the confidence in using it to treat people.’ (Participant 165, rotation 6, 2013)

### The rural environment

There were some students whose choice of a rural site was influenced by the accommodation and on-site hospitality. One such site was complimented for its comfortable accommodation, the three meals a day that were provided at no cost to the student and the fact that students were treated like doctors, based on information passed on by previous students. Other factors that influenced students were the support they would receive as a group if they were all based in one site together and an appreciation of the social learning in a primary health care setting. The opportunity to spend time with their primary or extended families was also mentioned.

‘The facilities available there, accommodation is clean and safe as well as safety throughout the block.’ (Participant 25, rotation 1, 2012)

‘Social, cultural + academic exposure. The fact that I will be able to master basic skills that will be important for my practise as a doctor next year.’ (Participant 182, rotation 7, 2013)

‘By word of mouth – previous groups have enjoyed their experience there. And it’s close to home – for when I need to do laundry and run out of food.’ (Participant 37, rotation 2, 2012)

Familiarity with the context was a consideration for some, as well as an opportunity to learn more about the facility.

‘I have relatives close to the site, this makes it a bit familiar to me in terms of social and environmental aspects.’ (Participant 113, rotation 4, 2013)

‘The facility is near my hometown, however I know absolutely nothing of it, and am only familiar with our Wits academic hospitals; Therefore I felt it imperative to learn about this hospital.’ (Participant 44, rotation 2, 2013)

A few students saw this as an opportunity to explore South Africa. Signing up for rural placement facilitated this and also provided them with a different clinical experience.

‘Nearing the end of our student years, keen for a different experience and exposure to rural setting. Also dynamics of working in a group and independence of not being at home.’ (Participant 186, rotation 7, 2012)

‘For the experience of a different environment.’ (Participant 197, rotation 7, 2014)

‘First time to site. Never been there. Excited!!!’ (Participant 36, rotation 2, 2012)

## Factors leading to an interest in urban placements

Factors that motivated the students’ preference of an urban site were identified as academic learning, environment, personal health, family responsibility and religion.

### Academic learning in an urban facility

Some students requested to be accommodated locally in undeserved sites in and around the Gauteng Province based on reports they have received from their peers about on-site tutorials and a structured learning approach in a particular site.

‘I wanted to stay in Gauteng. I heard good things about the site.’ (Participant 154, rotation 5, 2014)

‘Good team. Curiosity regarding Bertha Gxowa [an urban Gauteng hospital] … accessible to JHB [*Johannesburg*].’ (Participant 186, rotation 6, 2014)

‘Good range of clinical cases. Better supervision.’ (Participant 27, rotation 1, 2012)

‘Going to a peri-urban site to observe how non-academic institutions function.’ (Participant 190, rotation 7, 2012)

Within this group, some of the students were not comfortable with the format of the IPC block where activities are self-directed, supported by the local and academic supervisors.

‘Struggling with primary care approach … I am not competent enough.’ (Participant 201, rotation 7, 2013)

‘Proximity to Johannesburg. Exposure to a hospital on the internship group.’ (Participant 145, rotation 5, 2012)

An even smaller group of high achievers objected to rural placement based on perceived concerns that they were likely to lose their academic standing if they were in an unfamiliar territory.

‘Studying away from home … I know people who can help me around Joburg.’ (Participant 83, rotation 3, 2013)

‘Alex is a highly populated small rural settlement or informal settlement.’ (Participant 163, rotation 6, 2012)

‘Friends going with me; proximity to Johannesburg.’ (Participant 32, rotation 2, 2013)

‘Carrying a block, I needed to be close to prepare for integrated exams.’ (Participant 25, rotation 1, 2012)

### Personal health

The students in this category were considered to have special health needs and for that reason needed to be accommodated locally and often had permission from faculty.

‘Chronic illness – Crohn’s Disease. I need to be close to home and my specialist.’ (Participant 27, rotation 1, 2012)

‘Proximity to JHB. Suffer from a medical condition.’ (Participant 187, rotation 6, 2014)

### Family responsibility and financial challenges

Many students in this group cited family commitments as reasons for requesting local placement as they were either single parents or recently married with or without children.

‘It is within reasonable distance to home; because I am a mother to a 1 year old child; in case anything should happen at home.’ (Participant 149, rotation 5, 2013)

‘Close to home, not leaving my husband for 6 weeks.’ (Participant 118, rotation 4, 2014)

‘Family commitments (2 kids and preg wife), necessitated me being close to home.’ (Participant 129, rotation 4, 2012)

Others indicated that they did not have financial assistance and had to work part-time to finance their studies.

‘…Financial obligations do not permit me to work away from home as I am currently working to earn money to pay monthly loans and day to day living expenses.’ (Participant 442, rotation 7, 2013)

‘Financial matters – I have a student loan and need to pay monthly and I need to work to earn.’ (Participant 13, rotation 1, 2013)

### Urban environment

There were students who were not willing to venture out of Johannesburg. They cited relationships, comfort and access to resources as their main reasons.

‘Location. I wanted to be around Johannesburg I didn’t want to travel.’ (Participant 36, rotation 2, 2014)

‘Too old to share a house/accommodation with other students. Used to living independently. So I wanted a place in Joburg so I can stay at home.’ (Participant 134, rotation 5, 2013)

### Religion

A small subset of students objected to rural placements because of religious commitments, citing the fact that they were either fasting as a reason for choosing a Gauteng Province site or were on a special diet wherein certain cooking ingredients would not be available in a rural site.

‘I wanted to be able to live at home as I am fasting during this time. I also prefer my own accommodations.’ (Participant 202, rotation 7, 2014)

‘…Other responsibilities – work, family religious.’ (Participant 38, rotation 2, 2013)

## Ethical considerations

This study is part of an ongoing evaluation of the IPC block. The protocol for this was approved by the Wits Human Research Ethics Committee (M131162).

## Discussion

This study revealed that the rural platform is preferred by many medical students as it facilitates opportunities for them to gain confidence, manage the patient holistically, and is also considered to be a place to integrate learning and facilitate social and cultural competencies.^8,11^ Interestingly, the competency in speaking the local language was embraced as a benefit in the same way that it presented a challenge to those who were likely to be linguistically compromised by the setting.^13^ From this study, the factors that seem to have the greatest influence on the choice of rural placement, namely previous positive academic experiences and being of rural origin, are widely reported.^1,2,6,7,11,14,15^ Alternatively, it is the opportunity to have a different academic experience in a rural setting, and to be in a different location ‘to see more of South Africa’. Rose and Van Rensburg-Bonthuyzen make reference to a sense of belonging, socialisation and lifestyle factors as some of the factors that influence health care workers’ choice to remain in rural practice,^16^ so it is not surprising that these may be motivating factors for students. Similarly, being of rural origin does influence future practice as some of the students related their choice of site to their obligations of giving back to their own community, which fits with international evidence about the influence of rural origin.^1,2,9,11,14,15^ Previous research around the IPC rotation has noted that students value the opportunity to engage with communities and to understand their health needs, and this was echoed in the reasons for choosing rural sites mentioned in this study.^8,12^ An interesting finding is the extent to which medical students influenced their peers in choosing a rural site based on their experiences of the IPC block.

This study revealed that not all final year medical students are keen to take up placement in rural areas despite a progressive preparatory exposure from GEMP 1 to GEMP 3. An interesting tension of a kind can be seen in the students’ responses between those favouring a rural site from an academic perspective – they believe the learning will be better, because of the practical skills and context-based learning that occurs there – versus those who see the rural sites as important in terms of clinical practice and the contribution they may make, now and in the future. This ‘workforce versus education’ tension is a recognised issue in rural programmes internationally.^10^ This has been described particularly in longitudinal rural programmes.^17^ Therefore, it is interesting to see it reflected even in a short rotation such as IPC. It is important to hold this tension in balance; the challenge for rural placements is that if students all choose them only for one or the other reason, they will either be written off as not being academically sound (workforce focus) or will not achieve their desired long-term impact (education focus). Contextual factors were cited as the main reasons for seeking urban placement by the medical students who selected these. In contrast, contextual factors have been found to be some of the primary motivators that influence a health professional’s decision to work and stay in a rural area.^1,2,6,8,16^ However, the choices made by the students are supported by the fact that the majority of Wits students are raised and schooled in urban communities. In a study undertaken by Price and Weiner,^18^ 76% of Wits graduates surveyed had set up practice in urban-based settings, in line with a more recent study of career intentions of African medical students,^19^ thus supporting, albeit negatively, the association between childhood upbringing and affinity for rural practice.^15^ With only 21% of the class claiming to be of rural origin, limited exposure to public health and primary health care as career options and the majority of the undergraduate teaching being delivered by urban-based specialists, it is not surprising that Wits students favour urban-based medical practice.

There is no doubt that South Africa is dependent on its universities to produce the kind of graduate that will help alleviate the workforce challenges in rural areas.^1,2^ The fact that a number of students have to work to meet their financial needs calls for a review on the funding allocation to consider the country’s needs. In a similar study conducted in Nepal, the economic pressure was mentioned as one of the reasons students opted for an urban placement.^15^ It is possible that a number of students may be persuaded to take up rural placement in their final year if an offer is made to cover their tuition.^14^ This study has revealed a need for the society at large, including the medical fraternity, to support universities in their efforts to make rural practice a lot more appealing, especially to young doctors.

## Limitation

The study did not explore third-party influence on choice of site and this requires further exploration.

## Conclusion

Medical training presents unique challenges as academic staff have to balance personal and institutional interests with country needs. Although the primary responsibility of the academic coordinators is to deliver on the latter, some students have genuine reasons for objecting to rural placement that must be supported. It is incumbent on programme coordinators to identify ways to make rural placements more attractive and feasible for all students. Although the majority of Wits students are raised and schooled in urban settings, it is possible that early socialisation to primary health care settings may influence the number of students who would not have otherwise ventured out of Gauteng Province. At the same time, however, increasing the number of rural origin students selected into the medical programme, should also be pursued, as this is known to be a motivating factor for rural placement.^11^
